# “I take my pills every day, but then it goes up, goes down. I don’t know what’s going on”: Perceptions of HIV virological failure in a rural context in Mozambique. A qualitative research study

**DOI:** 10.1371/journal.pone.0218364

**Published:** 2019-06-17

**Authors:** Ivan Alejandro Pulido Tarquino, Emilie Venables, Jose Manuel de Amaral Fidelis, Ruggero Giuliani, Tom Decroo

**Affiliations:** 1 Médecins Sans Frontières, Tete, Mozambique; 2 Southern Africa Medical Unit, Médecins Sans Frontières, Cape Town, South Africa; 3 Division of Social and Behavioural Sciences, School of Public Health and Family Medicine, University of Cape Town, Cape Town, South Africa; 4 Health Provincial Directorat, Epidemiology Unit, Ministry of Health, Tete, Mozambique; 5 Médecins Sans Frontières, Maputo, Mozambique; 6 Institute of Tropical Medicine, Department of Clinical Sciences, Unit of HIV/AIDS & Infectious Diseases, Antwerp, Belgium; 7 Research Foundation Flanders, Brussels, Belgium; University of South Florida, UNITED STATES

## Abstract

**Background:**

HIV prevalence in Mozambique is estimated to be 13.2%. Routine viral load for HIV monitoring was first implemented in the rural area of Tete in 2014. Programmatic data showed an unexpected high proportion of high viral load results, with up to 40% of patients having a viral load above 1000 copies/ml.

**Objectives:**

This qualitative study aimed to explore perceptions about virological failure and viral load monitoring from the perspective of HIV positive patients on first-line antiretroviral therapy (ART) and health-care workers.

**Methods:**

The study was conducted in seven rural communities in Changara-Marara district, Tete province, Mozambique. A total of 91 participants took part in in-depth interviews (IDIs) and focus group discussions (FGDs), including health-care workers (n = 18), patients on ART in individual care or Community Adherence Groups (CAGs) who experienced virological failure and virological re-suppression (n = 39) and CAG focal points (n = 34). Purposive sampling was used to select participants. Interviews and FGDs were conducted in Nhuengue and Portuguese. IDIs and FGDs were translated and transcribed before being coded and thematically analysed.

**Results:**

Emergent themes showed that patients and health-care workers attributed great importance to viral load monitoring. A supressed viral load was viewed by participants as a predictor of good health and good adherence. However, some patients were confused and appeared distressed when confronted with virological failure. Viral load results were often little understood, especially when virological failure was detected despite good adherence. Inadequate explanations of causes of virological failure, delayed follow-up viral load results, repeated blood tests and lack of access to second-line ART resulted in reduced confidence in the effectiveness of ART, challenged the patient-provider relationship and disempowered patients and providers.

**Conclusion:**

In this rural context undetectable viral load is recognized as a predictor of good health by people living with HIV and health-care workers. However, a lack of knowledge and health system barriers caused different responses in patients and health-care workers. Adapted counselling strategies, accelerated viral load follow-up and second-line ART initiation in patients with virological failure need to be prioritized.

## Introduction

HIV still represents a major public health challenge. Unprecedented efforts, including decentralization of HIV services to primary health care level, have increased access to Antiretroviral Therapy (ART) [1]. Of the 19 million People Living with HIV (PLWH) in the East and Southern African region, 10 million are on ART [2]. However, poor adherence may result in Acquired Drug Resistance (ADR), and may hamper the effects of increased access to ART.

The HIV prevalence amongst adults in Mozambique (15–49 years old) is 13.2% [3]. Currently about half of this population is on lifelong ART [2]. Médecins Sans Frontières (MSF) has supported the Ministry of Health in providing HIV care in the central province of Tete since 2001.

Since 2008, patients on ART in Tete are able to choose between individual facility-based care, whereby they have to come monthly to the health facility to get a new ART refill, or Community ART Groups (CAGs). CAGs are peer groups with up to six patients on ART who take turns to fetch a monthly refill for the group at the health facility. Each CAG has a focal point, a group member who ensures proper functioning of the peer group. The CAG model has been described elsewhere in detail [4]. Attrition (patient death or loss to follow-up) was a major bottleneck during ART roll-out [5].

Routine viral load monitoring was first implemented in Tete in 2014. Samples are collected using the dried blood spot technique and sent to the HIV reference laboratory in the capital city of Maputo for analysis [6]. Results are then provided to individual health facilities by the reference laboratory. Identified patients with virological failure are contacted by HIV counsellors and receive two to three sessions of Enhanced Adherence Counselling (EAC), one session per month, in order to identify barriers to adherence, and together with health-care worker, find solutions. After six months from the first viral load they repeat the viral load test [7,8]. Those patients who have a high second viral load result are proposed to the National ART Committee to switch to second line of treatment when adherence to treatment is confirmed. Programmatic routine data showed an unexpected high proportion of high viral load results; up to 40% of the cohort had a detectable viral load above 1000 copies/ml in contrast with other low-and middle income countries which show virological suppression in more than 80% in patients over 12 months on treatment [9,10].

Previous quantitative and qualitative studies have documented barriers to adherence including long distances between communities and health facilities, transport costs, patient-health-care provider relationships, long waiting times at the health facilities, stigma, forgetfulness and work and family commitments which could also explain virological failure [11–13].

Although many qualitative studies have previously addressed barriers to adherence [14–17], including within this particular region [18–19], to the best of our knowledge no study has explored how patients and health-care workers perceive virological failure and routine virological monitoring in a rural area with difficult access to viral load testing.

## Methods

### Study design

This is a qualitative research study. Between July and August 2017, In-depth Interviews (IDIs) and Focus Group Discussions (FGDs) were conducted to explore patient and health-care worker perceptions of virological failure and viral load monitoring in the Changara-Marara districts of Tete province.

### Study setting

Changara and Marara districts consist of 15 health facilities, four in Marara and 11 in Changara. Of these, three clinics in Marara and nine clinics in Changara provide ART services. Clinical care is provided by medical doctors, medical technical officers and nurses. The 15 facilities are currently supported by MSF, and in June 2017 were providing ART to approximately 4223 active patients. In all 12 ART facilities, patients can choose between facility-based individual care or joining a CAG upon entry to the programme.

### Study population

Our study population was comprised of patients on ART in individual care or CAGs, and health-care workers. Participants in both categories were recruited from the two study sites of Changara and Marara districts. Community Adherence Group focal points and HIV Community activists, who are patients and who have experienced virological re-suppression, were also included in the sample. Health-care workers were included in the study as key informants in order to give a programmatic perspective on the research objectives based on their experience in providing ART services for the patients.

### Sampling and recruitment

A total of 91 participants were involved in the study, 54 from Changara and 37 from Marara. Purposive sampling was used for the patient group in order to select those who met the eligibility criteria of being adult patients having experienced virological failure and virological re-suppression. Where possible, a broad range of patients were selected to ensure that potentially diverse views, perspectives and experiences were included and presented. Patients were firstly identified in the routinely updated HIV cohort database, before being contacted by phone or face-to-face by one of the HIV counsellors working in the facility, who had been trained on the research study, and informed about the research. No participant was included in both an IDI and a FGD.

### Data collection

In-depth interviews and FGDs were conducted by the principal investigator (PI), with the assistance of a note-taker and a Portuguese and Nhuengue speaking translator. They were conducted using guides containing open-ended questions and probes. Guides were developed in English and then translated into Nhuengue.

During the IDIs and FGDs, participants were asked to identify themselves using a pseudonym. Before the start of each FGD the research team and the participants sat in a circle and a flipchart with ground rules was shared for approval by the group. Ground rules included active participation, that there were no wrong or right answers, confidentiality regarding data shared during the FGD and audio-recording.

The note-taker took note of the addressed themes and the general atmosphere. During a debriefing, conducted immediately after each IDI or FGD, the team met to discuss probes and saturation, and identified probes to be explored during the following IDIs and FGDs. Each IDI and FGD lasted between 60 and 75 minutes.

In order to mitigate the effect of a power imbalance between FGD participants, people with similar roles within facilities were grouped together where possible. Separate FGDs were subsequently organised for physicians, nurses and pharmacists; HIV counsellors and CAG focal points. Groups were not divided by gender as this was not deemed to be necessary in this context. Participants were given refreshments and their transport costs were reimbursed.

In-depth interviews and FGDs were held in local community locations which were chosen according to the participant’s preferences, taking into account privacy, ease of access and mitigating the risk of accidental disclosure. Commonly selected locations included churches, school classrooms, private homes and meeting halls.

### Data analysis

All interviews and FGDs were audio-recoded, transcribed verbatim and translated from Nhuengue into Portuguese if required. Through thematic content analysis [20], codes were identified, and organized manually into categories and major themes. The PI and one other co-investigator compared their findings and discussed areas of agreement as well as areas of divergence during interim and final analysis. Data analysis was conducted in Portuguese and selected quotes representing the identified codes and themes were translated into English. The findings of this study have been reported following the Consolidated Criteria for Reporting Qualitative Research (COREQ) guidelines [21].

### Ethics approvals

Ethics approval was granted by the Médecins Sans Frontières Ethics Review Board Geneva, Switzerland and the National Ethics Review Board in Mozambique, the *Comité Nacional de Boiética para a Saúde (275/CNBS/17)*. Written informed consent for participation to the study was obtained from all participants prior to data collection.

## Results

A total of 39 IDIs were conducted in seven different communities across both districts. Eight FGDs were held, three with health care workers (n = 18) and five with CAG focal points and an HIV activist (n = 34). A total of 39 patients (15 male and 24 female), including those in individual care and CAGs participated in interviews. Of these, 56% (22/39) had experienced virological failure and 44% (17/39) virological re-suppression. In addition, 18 health-care workers from ART sites in both districts took part in FGDs. Focus Group Discussions were also conducted in both districts with 34 CAG focal points and one community HIV activist.

The demographic characteristics of the study participants are shown in Tables 1 and 2.

**Table 1:** In-depth interviewee characteristics.

District	Community	Number of interviewees	Sex		Age	Number of years on ART	CAG	VF^ǂ^	VR^#^
			Male	Female	Median (range)	Median (range)			
			N (%)	N (%)			N (%)	N (%)	N (%)
**Marara**	**Matambo**	7	1 (14.3)	6 (85.7)	41.8 (32–50)	5 (2.3–7.7)	5	5	2
	**Marara centro**	5	1 (20.0)	4 (80.0)	46 (30–57)	4.9 (3.8–8.3)	3	3	2
	**Cachembe**	5	4 (80.0)	1 (20.0)	48.6 (47–49)	6.9 (2.9–11.8)	5	5	0
**Changara**	**Missawa**	4	2 (50.0)	2 (50.0)	38 (26–44)	2.5 (1.3–4.8)	2	2	2
	**Mazoe**	4	1 (25.0)	3 (75.0)	33 (31–35)	3.8 (1.4–5.2)	2	1	3
	**Changara**	6	3 (50.0)	3 (50.0)	44.5 (28–62)	6.6 (1.4–9.5)	5	3	3
	**Dzunga**	8	3 (37.5)	5 (62.5)	40.3 (24–48)	5.7 (2.5–9.3)	5	3	5
**TOTAL**		39	15 (38.5)	24 (61.5)	41.7 (24–62)	5.3 (1.2–11.8)	27	22	17

ǂ Virological Failure

# Virological Re-suppression

CAG: Community ART Group; ART: antiretroviral therapy

**Table 2:** Characteristics of Focus Group Discussion participants.

District	Number of FGDs	Type of participants	Average number of participants	Sex		Health-care workers				Patients
						Medical officer	Nurse	Counsellor	Pharmacist	CAG FP
				*Male*	*Female*					
				*N*	*N*	*N*	*N*	*N*	*N*	*N*
**Marara**	2	CAG Focal Points*	6	6	6	0	0	0	0	12
	1	Health-care workers	6	2	4	2	3	0	1	0
**Changara**	3	CAG Focal Points	6	5	17	0	0	0	0	22
	1	Health-care workers	6	3	3	3	3	0	0	0
**Tete**	1	HIV counsellor	6	2	4	0	0	6	0	0
**TOTAL**	8			18	34	5	6	6	1	34

*Included one HIV activist

CAG: Community ART Group; CAG FP: CAG Focal Point, N: number; FGD: focus group discussion

### Viral load and health status

Viral load results were perceived to be very important by most of the patients, and were considered as a marker of their general health status. As one patient stated, viral load results help to *“know what is going on in the body*, *to see if I’m taking the pills well*, *if it results in an effect*, *or it’s going up*, *or it’s going down”* (IDI 30; *47 year old male patient*).

Some patients perceived viral load testing as mandatory but also beneficial:

*“Because of the suffering that I’m experiencing*, *I have to subject myself, otherwise I would be dead, isn’t it? It’s good” (IDI 8; 47 year old female patient)*

*“Usually they [HCWs] call us to come to the health centre and collect our blood and then they show us [our results]” (IDI 16; 32 year old female patient)*.

A *few* patients referred to viral load testing as a tool to monitor the ‘quantity’ of the virus in their blood:

*“I want to know the quantity … When I will know it*, *then it’s a reason for me to ask you to help me to show me how I can reduce my load.” (IDI 17; 24 year old female patient)*

### Patient perceptions and explanations of virological failure:

Patients had different perceptions of what virological failure meant. Many of them understood it as a failure related to irregular pill intake, witchcraft or unprotected sexual relationships. Being notified about virological failure can provoke intense sadness and thoughts of despair and death. In one example, the patient talked about how the virus is ‘*waking up’*:

*“If you delay one hour [to take your pill]*, *that hour it [the virus] is awake … It eats you at that very moment.” (IDI 18; 30 year old man patient)*

Patients feel confused when they are diagnosed with virological failure if they report being adherent to their daily treatment.

*“Sometimes I’m worried*, *I take the pills but the viral load is increasing, this worries me.” (IDI 12; 26 year old female patient)*

Some patients with virological failure were reported to question whether the drugs are still effective, by asking if ‘*the pills are good or not*” *(FGD 5; Counsellor)*. Some reportedly want to stop treatment, as the drugs do not seem to be having an effect:

*“And also it may create a problem, the patient may want to stop his treatment*. *Some patients even say “if it’s like this, then I prefer to stop.” (FGD5; Counsellor)*

Most patients reported being shocked or saddened when they were told by health care providers that their results showed virological failure.

*“I take my pills every day, but then it goes up*, *goes down; I don’t know what’s going on. It hurts my heart, because even though I do what they [health care providers] tell me, my heart hurts.” (FGD3; CAG Focal Point)*

*“Sometimes I feel worried*, *I am taking my pills but the viral load is increasing, this worries me, what is happening? I am taking my medicine correctly, and my viral load is increasing.” (IDI 12; 26 year old female patient)*

Virological failure was also associated with death by some patients. This nurse described how one of her patients believed that virological failure meant that her life was ending: “*now it’s going up*, *thus my life will end soon* … *I’m saying farewell to you*.” (FGD 1*; Nurse*)

Another interviewee described how she dreamed of death after she was notified of virological failure:

*“When I sleep*, *I end up dreaming of an urn, a coffin next to me. When I wake up I do not see anything, I’m always asking myself why this is happening to me.” (IDI 6; 30 year old female patient)*

Others patients such as the one cited below, believed that virological failure was a result of witchcraft that may affect her pill intake, leading to treatment failure:

*“They [people in the community] do that work of witchcraft*, *sorcery… They start putting on you other things and then all gets worse… How they do that exactly, I do not know, but now I do have that thing in my mind that this person can do me harm, and I get scared. Thus it [viral load] gets worse and at the end people start thinking that they are not taking the medicine correctly, and everything becomes complicated.” (IDI 16, 32 year old female patient)*

Explanations of virological failure provided by patients include non-adherence, which in turn is often explained by a lack of regular or timeous pill intake, alcohol use, or being on treatment for an extended period of time. Some associate virological failure with betrayal and a lack of condom use when having sexual relationships “*outside*” of their relationship.

*“Virological failure can be related to my boyfriend*, *because when we don’t have sex, everything goes well, but when we have sex, the viral load goes up immediately.” (IDI 19, 44 year old female patient)*

Patients and health-care workers report that they have heard about people who are not adhering well to treatment and who throw their pills away or store them at home. They report that supplies of ART can be found in the houses of those who become sick and die.

*“Some even throw away [their pills] in the latrine*; *sometimes they throw them away in the neighbourhood … Little children play with those medicines. Sometimes they give them to other people and say "I'm not sick, go, you take them.” (FGD 3, CAG Focal Point)*

“There are patients who always come. They do not fail to pick up their medicines, but when they get the drugs, once at home, they do not take them. They keep saving them, they keep saving them. When he dies, we take him [his body] to the funeral, and then we see so many bottles of the medicines [in his house].” (FGD1, CAG Focal Point)

Some patients discussed how their viral load remained high despite their efforts to improve adherence, and health-care workers explained that they do not understand why some patients present with virological failure. They described being puzzled that despite apparent good adherence after counselling, some patients still had high viral load results.

In such cases, health-care workers suspect the patient was infected with a virus that was already resistant, and they proposed a second-line treatment regimen. When health-care workers introduce second-line ART and the patient has a re-supressed viral load, they conclude the patient was adherent throughout.

*“There is a family*, *a mother with her children, they are all on treatment, but they all had a high viral load. They had several counselling sessions. We visited them, to change their behaviour, but she is taking her pills, she is taking them well. She had shifted her line of treatment, the younger son too…and the other daughter too. They are saying that they are taking their pills. Then sometimes we think that these people already at the beginning, they got a resistant virus. Now, [that they are] already on second line, they had an undetectable viral load.” (FGD 5; Counsellor)*

### Viral load procedures: Lack of ownership

Patients are informed about the procedures for viral load testing by health-care workers, but viral load is often perceived as something unfamiliar that they do not always have ownership over. In addition, patients do not always fully understand the flow of viral load testing, with one patient referring to the reference laboratory system with the words “*they have their machines over there in Maputo [the capital]*, *they know what to do*”. Nevertheless, viral load is perceived as a marker of their own adherence to pill-intake.

“*They say that we are providing a blood sample that they can send to the white [people] to look for something*, *for them to do the analysis*, *to be able to see if it’s going up or not*, *the viral load*.*” (IDI 12; 26 year old female patient)*

*“They know where they are sending it*, *when they have the result they call us, they say yes, you are adherent or not.” (IDI 16; 32 year old female patient)*

Patients did not always fully understand the procedures for viral load testing and did not always feel a sense of ownership over the process:

*“It is important to do a [test]*, *because as they have machines, they are able to tell if it’s going well like this … But by myself, I don’t know anything.” (IDI 15, 33 year old male patient)*

### Delays in obtaining viral load results, and the patient-provider relationship

Patients and health-care workers alike complained about the delay between blood samples being taken and viral load results being ready. Health-care workers discussed how they need the results to decide whether a patient needs to start second line treatment, but also described how the results sometimes arrived when the patient’s condition had worsened, or even when the patient had died.

*“We could have been in the month of March*, *and today we are in August and still it did not appear … We then propose second line [treatment]. When the result arrives we have already lost the patient …died …because the viral load result was delayed a lot.” (FGD 1; Nurse)*

Some health-care workers would like to do a viral load test earlier, when the patient has been on ART for three months, to ensure a follow-up viral load, if needed, and detect failure earlier.

*“If we did it [viral load testing] a bit earlier*, *if we could do it at 3 months treatment, just for us to understand how we are doing in terms of patient adherence. After six months we could wait for the second viral load, to see which barriers this patient has, and if they were overcome.” (FGD 2; Medical officer)*

The delay in obtaining viral load results can reduce patient trust in their clinicians. Clinicians, such as the medical officer cited below, described how he apologised for the delays, and the need to negotiate another blood sample.

*“Sometimes we have cases that we lose because viral load tests are delayed and we were waiting for it … We apologize to our patients [for the delay] until we’re tired and sometimes they treat us like liars*: *“you always say that the result will arrive soon…” Thus we are compromised …” (FGD 1; Medical officer)*

Patients reported frequently having multiple blood samples taken to obtain a single viral load result. In one FGD, a health-care worker described how a patient asked if their blood was being sold *“we are always here*, *giving blood*, *actually*, *are you selling our blood*?*”* (FGD 5; Counsellor)

## Discussion

To the best of our knowledge, this is the first qualitative study on perceptions of virological failure and viral load monitoring in a rural African setting. Our findings revealed a range of perceptions about the meaning of virological failure, viral load monitoring practices and their effect on treatment adherence and the relationship between provider and patient.

Patients gave great importance to having a viral load test and receiving the results. We found that an undetectable viral load was meaningful for patients re-suppressing their viral load. It is synonymous with good health, and enables them to continue with their daily lives and activities. Indeed, perceived treatment efficacy is since long recognized by previous research as an important motivating factor in chronic disease care [22, 23].

On the other hand, many of the patients in the study perceived virological failure as a serious issue that put their general health at risk. Within the informal social networks of PLHIV in the study districts (such as the networks that form within CAGs), many patient stories are shared about non-adherence resulting in illness and then death. Patient stories find their origin in events that are witnessed in the community, and messages shared by counsellors and clinicians.

A high viral load result is perceived as a shock, can provoke intense sadness and a feeling of loss, and may lead to feelings of depression or a loss of confidence in their ability to manage their own health. Previous studies have identified depression as a predictor of virological failure [24]. However, we believe that this relationship may be even more complex, and patients who are diagnosed with virological failure may then have difficulties coping with this information and become depressed as a result. In some cases, as described above, the diagnosis of virological failure led to bad dreams. Therefore, when patients are confronted with a laboratory result showing virological failure, some consider it as a sign of an inevitable death. The general belief in a bidirectional relationship between the health status and the mind, and spirituality has been documented before [25].

As may be expected, we observed that patients used a different kind of language from the health-care workers providing the services when talking about their viral load results and virological failure. Health-care workers used language characterized by biomedical terms, whereas patients seemed to think and talk using symbols, such as the virus ‘waking up’. Likely, messages shared during counselling sessions may be not completely understood by patients. Counselling has been recognized as an important enabler of HIV service utilization [13] however, how messages can be translated to fit best the local socio-culturally context merits more research [12]. Moreover, key counselling messages focus on daily pill intake and the consequences of poor adherence. Counsellors and patients described the challenges associated with providing an explanation of virological failure in patients with good adherence. Health-care workers and patients were little prepared for the paradoxical situation in which good adherence (or improved adherence after enhanced adherence counselling) is not accompanied by an undetectable viral load. Counselling will have to adapt as service delivery evolves [26]. Looking for a cause for virological failure, patients explained virological failure in different ways, such as witchcraft or their partner’s sexual practices, especially if the partner is believed to have sexual relationships outside the marriage. Health-care workers and counsellors emphasized the patient’s responsibility in taking pills daily to control their virus but did not always understand the circumstances under which viral load would be high, regardless of adherence.

Only a few of the patients in our study fully understood the viral load monitoring procedures and that viral load monitoring was a tool that could help them to take control of their HIV infection. Most described blood collection as a practice that must be done, without having a clear idea of how the results can help them to manage better their condition. Patients gave great importance to having a viral load test and receiving their test results. We found that an undetectable viral load had a very positive meaning for patients who had a re-suppressed viral load. We speculate that an undetectable viral load is perceived as a sign of good health, which enables them to continue with their treatment and continue with their daily lives and activities. In addition, patients are less likely to adhere to a drug they perceive as ineffective [27] [22]. The incomplete understanding of virological failure may explain the adverse effects of high viral load results on the motivation to adhere.

Moreover, most patients do not understand how the blood sample chain works, as samples are sent by plane to the capital city of Maputo, and results are then sent to individual clinics by email. On the one hand it is a distant intervention that they cannot control, whereas on the other hand the results can have a profound impact upon their life [23]. Some patients even expect the test to tell them if they are taking their pills correctly or not. As results can arrive with significant delays, or may not arrive at all, patients are frequently requested to provide new blood samples. Dissatisfaction challenges the provider-patient relationship and leads some patients to question if their blood is being used for other purposes than viral load testing [13,28]. Consequently, some patients felt disempowered, which may affect negatively their motivation to adhere [12]. Providing viral load testing closer to patient’s homes may demystify the process and be a good example of a patient-centred approach to HIV care [29].

Clinicians may also be concerned or confused when patients have a high viral load result despite apparent good adherence, especially if this was verified through home visits. However, as clinicians become more familiar with the concept of resistance, including acquired and transmitted resistance, they wish to shift patients with virological failure onto second line treatment. However, they are confronted with delays when they request a follow-up viral load, which can put health-care workers in a very difficult position. They are committed to ensuring that patients receive proper clinical care and the appropriate treatment regimen, but are also reliant on the limited resources of the national health system. Given that confidence is essential for an effective patient-provider relationship, [22,30] it is concerning that there could be a lack of trust. Moreover, clinicians repeatedly reported that such delays often resulted in a worsening clinical condition for the patient, and even death. As a result of this, care providers can also feel disempowered and unable to help their patients. Previous research on viral load monitoring in Mozambique’s capital showed that only one in three of those with a high (≥3500 copies/ml) viral load had a follow-up viral load test, and that only one in three of those with a high follow-up viral load started second-line ART [31]. When programs opt for routine viral load monitoring, there should be a clear and functional plan for those identified as failing.

The present study has several limitations. Interviews were not conducted with patients who were considered lost to follow up, although they may have had a different perception of virological failure or viral load monitoring to our study population. Secondly, the themes identified by the present study are dependent to the extent the interviewees disclosed topics they found important or relevant to share. Thirdly, over half of the interviews were conducted in local Nhuengue by a non-Mozambican researcher with the assistance of a Mozambican translator, which may have caused some cultural and language barriers and affected the level of disclosure of the interviewee. Finally, since IDIs and FGDs were conducted in a very rural context, findings may not be generalizable to urban contexts. Despite these limitations, the present study has some important strengths. First, at the end of every interview emerging codes were identified and saturation was discussed. Secondly, formal interim analysis and final analysis was conducted independently by two investigators, who discussed areas of agreement and divergence. Given that qualitative research is not designed to test a hypothesis or determine the frequency of events, the themes that emerged in this study may inform future research. Moreover, additional qualitative studies are needed to explore experiences with adherence strategies in patients with virological failure.

## Conclusions

Even though our findings are not generalizable to other rural contexts, this qualitative study on the perceptions of virological failure and viral load monitoring in rural Mozambique showed that most patients and health-care workers placed great importance to viral load testing. Structural delays of the actual system in obtaining viral load results also made virological failure difficult for clinicians to manage and in turn made patients and clinicians felt frustrated and disempowered.

Counselling strategies should be adapted to the local context, and should include clear messages for different scenarios, including virological failure in patients reporting good adherence. Viral load monitoring should be coupled with a clear plan to start those identified as failing on second-line ART.
